# Co-inoculation of the endophytes *Bacillus thuringiensis* CAPE95 and *Paenibacillus polymyxa* CAPE238 promotes *Tropaeolum majus* L. growth and enhances its root bacterial diversity

**DOI:** 10.3389/fmicb.2024.1356891

**Published:** 2024-02-23

**Authors:** Isabella Dal’Rio, Eliene dos Santos Lopes, Karen Caroline Ferreira Santaren, Alexandre Soares Rosado, Lucy Seldin

**Affiliations:** ^1^Instituto de Microbiologia Paulo de Góes, Universidade Federal do Rio de Janeiro (UFRJ), Rio de Janeiro, Brazil; ^2^Bioscience, Biological and Environmental Sciences and Engineering Division (BESE), King Abdullah University of Science and Technology (KAUST), Thuwal, Saudi Arabia

**Keywords:** *Tropaeolum majus*, *Bacillus thuringiensis*, *Paenibacillus polymyxa*, biofertilization, plant growth-promoting bacteria

## Abstract

*Tropaeolum majus* L. is a versatile edible plant that is widely explored due to its medicinal properties and as a key element in intercropping systems. Its growth could be improved by the use of biofertilizers that can enhance nutrient uptake by the plant or provide tolerance to different abiotic and biotic stresses. In a previous study, 101 endophytes isolated from *T. majus* roots showed more than three plant growth-promoting (PGP) features *in vitro*, such as phosphate mineralization/solubilization, production of siderophores, antimicrobial substances and indole-related compounds, and presence of the *nifH* gene. To provide sustainable alternatives for biofertilization, the genomes of two promising endophytes—CAPE95 and CAPE238—were sequenced to uncover metabolic pathways related to biofertilization. Greenhouse experiments were conducted with 216 seeds and 60 seedlings, half co-inoculated with the endophytes (treatment) and half inoculated with 1X PBS (control), and the impact of the co-inoculation on the plant’s bacteriome was accessed through 16S rRNA gene metabarcoding. The strains CAPE95 and CAPE238 were taxonomically assigned as *Bacillus thuringiensis* and *Paenibacillus polymyxa*, respectively. Metabolic pathways related to the enhancement of nutrient availability (nitrogen fixation, sulfate-sulfur assimilation), biosynthesis of phytohormones (indole-3-acetic acid precursors) and antimicrobial substances (bacilysin, paenibacillin) were found in their genomes. The *in vivo* experiments showed that treated seeds exhibited faster germination, with a 20.3% higher germination index than the control on the eleventh day of the experiment. Additionally, treated seedlings showed significantly higher plant height and leaf diameters (*p* < 0.05). The bacterial community of the treated plants was significantly different from that of the control plants (*p* < 0.001) and showed a higher richness and diversity of species (Chao and Shannon indexes, *p* < 0.001). A higher relative abundance of potential synergistic PGP bacteria was also shown in the bacteriome of the treated plants, such as *Lysinibacillus* and *Geobacter*. For the first time, co-inoculation of *B. thuringiensis* and *P. polymyxa* was shown to have great potential for application as a biofertilizer to *T. majus* plants. The bacterial consortium used here could also be explored in other plant species in the future.

## 1 Introduction

*Tropaeolum majus* L. is a versatile plant originated from Latin America. *T. majus* is an edible plant that has medicinal properties and is commonly used for landscaping. Its leaves and flowers are usually consumed in salads, its seeds are pickled, and its roots can be used for tea consumption. *T. majus* is also relevant for intercropping systems since it is tolerant to severe drought stress, is able to improve soil coverage and prevent the growth of weeds on organic farms, and is usually cultivated in a consortium system with other crops (Golian et al., 2023; Mircea et al., 2023). Moreover, the plant extracts show great antimicrobial activity, can inhibit the growth of cancer cell lines, have antiadipogenic effects and have also been studied as a possibility for acute bronchitis treatment (Ailane et al., 2022; Vrca et al., 2022; Albrecht et al., 2023).

In a previous study, the root bacteriome of *T. majus* plants grown in an organic farming system was analyzed, and 236 endophytes were isolated from the plant roots (Dal’Rio et al., 2022). Among the isolates, the two strains *Bacillus* sp. CAPE95 and *Paenibacillus* sp. CAPE238 (denoted previously as E95 and E238, respectively) showed five plant growth-promoting (PGP) traits *in vitro*. These features included mineralization and solubilization of phosphate, biosynthesis of siderophore and antimicrobial substances, production of indole-related compounds (exclusively in the *Bacillus* sp. CAPE95 strain) and the presence of the dinitrogenase reductase subunit (*nifH*) encoding gene (exclusively in the *Paenibacillus* sp. CAPE238 strain).

Both *Bacillus* and *Paenibacillus* spp. are ubiquitously present in the environment, and they are commonly found in close association with plants. These genera include endospore-forming bacteria that may possess PGP traits, as they can enhance plant nutrient uptake, elicit immunity, alleviate abiotic stresses and act as biocontrol agents through the production of antimicrobial substances (Langendries and Goormachtig, 2021; Soni and Keharia, 2021). The ability of these genera to produce endospores gives them a better chance to colonize the plant and promote growth, since they can easily disperse in the environment and resist harsh conditions, such as salinity and drought. For this reason, we believe that endospore-forming bacteria are more appealing for the development of biofertilizers than other plant growth-promoting bacteria (PGPB).

Biofertilizers, also known as microbial inoculants, are organic products composed of microorganisms that are able to colonize plants and promote their growth by enhancing plant nutrient uptake and protecting them against phytopathogens and pests. Moreover, these PGP microorganisms can also produce lytic enzymes that contribute to reducing the toxicity in soil caused by chemical fertilizers (Kour et al., 2020). In this context, the use of biofertilizers in medicinal plants has been proven to increase the quality and quantity of secondary metabolites that can be extracted and applied by various industries (Ahmad et al., 2022). For example, these metabolites can be a source of phytomedicines relevant for pharmaceutical industries.

To further investigate the potential application of the endophytes *Bacillus* sp. CAPE95 and *Paenibacillus* sp. CAPE238 as biofertilizers, the whole genomes of these strains were explored, leading to a taxonomic classification at the species level, and the metabolic pathways associated with biofertilization were identified. Moreover, the two strains were co-inoculated into *T. majus* seeds and seedlings to evaluate their biofertilization potential *in vivo*. The impact of the consortium inoculation on the plant bacteriome was assessed through 16S rRNA gene metabarcoding, and the enrichment of bacterial taxa was investigated. The results obtained in the present study provide evidence that the selected endophytes have great biotechnological potential for application in agriculture.

## 2 Materials and methods

### 2.1 Bacterial strains

The endophytes *Bacillus* sp. CAPE95 and *Paenibacillus* sp. CAPE238 were previously isolated from *Tropaeolum majus* L. surface-sterilized roots of plants cultivated in an organic farming system in Nova Friburgo, Rio de Janeiro, Brazil (Dal’Rio et al., 2022). The strains were stored in tryptic soy broth (TSB) supplemented with 20% glycerol at −80°C, and to perform the DNA extraction and *in vivo* assays, the strains were reactivated in 50 mL of TSB for 24 h at 32°C under agitation (150 rpm).

### 2.2 Genomic analysis

#### 2.2.1 DNA isolation and whole genome sequencing

Both strains CAPE95 and CAPE238 had their DNA isolated using the methods described by Rosado and Seldin (1993). The bacterial cultures of each strain were centrifuged (10,000 × *g* for 15 min), concentrated in 5 mL of Tris-EDTA-NaCl buffer (pH = 8.0), treated with 500 μL of lysozyme (10 mg mL^−1^ for 1 h at 37°C) and 500 μL of 10% sodium dodecyl sulfate (10 min at 37°C). The DNA purification process was performed as described by Seldin and Dubnau (1985). The concentration and purity of the DNA were determined using a Quibit^™^ 3.0 fluorometer (Thermo Fisher Scientific^™^, Waltham, MA, United States) and a Nanodrop spectrophotometer (Thermo Fisher Scientific^™^), respectively, guaranteeing a total genomic DNA concentration ≥ 200 ng, in a sample volume ≥ 20 μL and adopting the parameters of purity of A260/280 = 1.8–2.0. Furthermore, the DNA from both strains was sent to Novogene (SAC, United States). Paired-end libraries (2 × 150 bp) with a 350 bp insert size were constructed, and the whole genome was sequenced on an Illumina NovaSeq 6000 following the manufacturer’s recommendations.

#### 2.2.2 *De novo* genome assembly

An initial quality control step of the raw reads obtained from both the CAPE95 and CAPE238 genomes was performed using fastp (Chen et al., 2018) to remove adapters and low-quality bases. Furthermore, the quality of the reads was checked with FastQC (Andrews, 2010), and MultiQC (Ewels et al., 2016) was used to merge the results from both fastp and FASTQC softwares. The 150 bp paired-end reads of each strain were assembled using Unicycler (Illumina-only assembly method; Wick et al., 2017), and the quality of the assembled genomes was accessed using QUAST (Gurevich et al., 2013).

#### 2.2.3 Taxonomic classification and functional annotation

To determine the taxonomic classification of each strain, GTDB-Tk v2 software (Chaumeil et al., 2022) was used. Briefly, GTDB-Tk uses Prodigal (Hyatt et al., 2010) for gene calling, HMMER (Finn et al., 2011) for the identification of 120 marker genes and alignment in the database Genome Taxonomy Database (GTDB) and pplacer (Matsen et al., 2010) for domain-specific tree construction. ANI (Average Nucleotide Identity; FastANI calculated; Jain et al., 2018) was used for genomic distance calculations, whereas the threshold to be classified as the same species was mostly considered ANI ≥ 95% (Olm et al., 2020). The generated phylogenomic tree was exported to iTOL v6,^1^ and metadata were added.

Moreover, as species assignments within the *Bacillus cereus* group can be intricate (Carroll et al., 2022), the *Bacillus cereus* cgMLST (core genome multilocus sequence typing; Tourasse et al., 2023) and rMLST (ribosomal multilocus sequence typing; Jolley et al., 2018) tools—available at PubMLST^2^—were also used for more accurate classification of the CAPE95 strain.

The software Anvi’o (Eren et al., 2021) was used for functional annotation via the Kyoto Encyclopedia of Genes and Genomes (KEGG) database for genes and metabolic pathways (Release 107.0, July 1, 2023; Kanehisa et al., 2023). Comparatively, functional annotation was also performed with Prokka (Seemann, 2014), and the results were analyzed using UniProt UGENE (Okonechnikov et al., 2012).

### 2.3 Greenhouse *in vivo* experiments

#### 2.3.1 Seed germination test

The reactivated strains CAPE95 and CAPE238 were grown in TSB with the addition of 1.5% agar (TSA) to confirm their purity by colony morphology visualization and through the Gram staining technique. Moreover, 200 mL of sterile TSB was inoculated with 1% of the previous growth of each strain and incubated under the same conditions as those described in section 2.1. Subsequently, the optical density of each bacterial culture was adjusted to 10^8^ CFU mL^−1^ (OD600 = ~1.0), and the cells were centrifuged for 15 min at 8,000 rpm. The supernatant was discarded, the cell pellet of each strain was resuspended in 200 mL of 1X sterile phosphate-buffered saline (PBS), and the suspensions of each strain were mixed to obtain the consortium. To inoculate the seeds, 100 mL of the consortium suspension was added to 900 mL of sterile 1X PBS, yielding a suspension of 10^7^ CFU mL^−1^.

In the greenhouse located at UFRJ, Rio de Janeiro, Brazil (22°50′28″S, 43°14′5″W), 1.2 L pots containing 1 L of Carolina soil substrate were added with half of the suggested concentration of the fertilizer Forth Cote 14-14-14 (2.5 g L^−1^). Each pot was sown with three *T. majus* seeds, and each seed was superficially inoculated with a Pasteur Pipet with 2 mL of the treatment or the control (sterile 1X PBS). Afterwards, the pots were distributed into four blocks, each containing control (*n* = 9) and treatment (*n* = 9), totaling 36 vases for each condition (control or treatment), with 108 seeds for each condition. Seed germination of *T. majus* usually occurs between 10 and 20 days; therefore, the experiment was conducted for 21 days. The pots were irrigated 3 times a day for 3 min. The germination index of the seeds was calculated for each condition (control and inoculated with the two strains) by dividing the number of total germinated seeds by the total number of seeds (108 for each condition).

#### 2.3.2 Seedling growth promotion test

The consortium suspension was prepared as described previously, except that the suspension was maintained at 10^8^ CFU mL^−1^ (OD600 = ~1.0). In the greenhouse, 30 days after the seeds were sown, seedlings were thinned to obtain only one per vase. This time, three blocks were established, maintaining only the seedlings that showed satisfactory growth (i.e., those that showed similar heights and numbers of healthy leaves) for 30 days. These seedlings were then redistributed to either the treatment or control, keeping the same initial condition of each plant, and maintaining 10 replicates for each condition (treatment or control) in three different blocks (a total of 30 replicates per condition).

The treatment (CAPE95 + CAPE238) and the control (sterile 1X PBS) were inoculated by foliar spray (approximately 10 mL per plant). The experiment was set for 21 days, and plant parameters were registered through time—number of healthy leaves, percentage of chlorosis, average leaf diameter and plant height. The average leaf diameter was calculated using the average diameter of five different leaves of each replicate, and the chlorosis percentage was calculated by the number of yellowing leaves divided by the total number of leaves and multiplied by 100. The metrics were measured 7, 14, and 21 days after inoculation.

### 2.4 16S rRNA gene metabarcoding

#### 2.4.1 Total DNA isolation from *Tropaeolum majus* roots

At the end of the experiment in the greenhouse (~60 days after sowing the seeds), three replicates of each condition (treatment or control) from each of the three blocks were sampled, totaling nine plants for the control and nine plants for the treatment. Each plant was carefully removed from the pots to avoid disrupting the thinner roots, and they were placed in sterile plastic bags and immediately transported to the laboratory.

The aerial part of the plant was cut with sterile scissors, and the roots were shaken to remove the loosely attached soil. Ten grams of roots with adhered soil from each of the 18 plants were weighed and homogenized with 5 mL of sterile distilled water in a sterilized mortar and pestle, in which they were macerated to obtain an extract of rhizosphere and endophytic bacteria. DNA extraction was performed as described in Dal’Rio et al. (2022).

#### 2.4.2 Metabarcoding sequencing and analysis

The total DNA extracted from the roots of eighteen *T. majus* plants (treatment and control) was sent to Novogene (Sacramento, CA, United States) and sequenced using the Illumina NovaSeq 6000 platform. The pair of primers 799F (5′-AACMGGATTAGATAC CCKG-3′) and 1193R (5′-ACGTCATCCCCACCTTCC-3′) for regions V5-V6-V7 of the *rrs* gene, encoding the 16S rRNA gene (Beckers et al., 2016), was added with barcodes to amplify fragments of approximately 394 bp. Paired-end sequencing libraries (2 × 250 bp) were constructed. After sequencing, the raw reads were filtered by removing primers and barcodes to obtain high-quality sequences. The sequences obtained from the 18 samples were analyzed with Mothur v.1.48.0 (Schloss et al., 2009), following the standard operating procedure described by Kozich et al. (2013), accessed in June 2023.^3^

The results were exported to PAST 4.02 (Hammer et al., 2001), where boxplots were constructed for alpha-diversity analysis and a nonmetric multidimensional scaling (NMDS) analysis was performed for beta-diversity using the Bray Curtis dissimilarity index. To explore the differential relative abundance of taxa, heat trees were constructed using the metacoder package (Foster et al., 2017) from RStudio version 2023.06.0 + 421 (RStudio Team, 2020) and the core bacteriome—the most abundant bacterial taxa common in all replicates of each condition—was accessed using the tool MicrobiomeAnalyst 2.0 (Lu et al., 2023).

### 2.5 Statistical analyses

PAST 4.02 software (Hammer et al., 2001) was used to perform the statistical analyses of the greenhouse experiments and the metabarcoding analysis. For the greenhouse experiments, the plant growth parameters measured were initially checked for normality (Shapiro–Wilk normality test, *p* > 0.05) and homoscedasticity (Levene’s test, *p* > 0.05), so a two-way ANOVA could be performed, considering the influences of the conditions (treatment and control) and the influence of the different blocks. Significant differences were considered assuming degrees of freedom of 10% (*p* < 0.1), 5% (*p* < 0.05) and 1% (*p* < 0.01). The metabarcoding diversity indexes were analyzed as described by Dal’Rio et al. (2022). For the relative abundance of taxa, the Wilcoxon test was used (*p* < 0.05) in the software RStudio version 2023.06.0 + 421 (RStudio Team, 2020).

## 3 Results

### 3.1 Genome analyses

#### 3.1.1 Taxonomic assignment of the strains CAPE95 and CAPE238

The genome sequences of both strains are deposited in the GenBank database under the accession numbers JAWPHG000000000 (CAPE95) and JAWPHF000000000 (CAPE238) in BioProject PRJNA1033832. The genome of strain CAPE238 has 5,767,258 bp, with a G + C content of 45.53% and a total of 5,138 coding DNA sequences (CDS). The genome of strain CAPE95 has 5,655,448 bp, with a G + C content of 34.85% and 5,537 coding DNA sequences (CDS). The full report of the assembly of the two genomes is provided in Supplementary Table S1.

**Figure 1:**
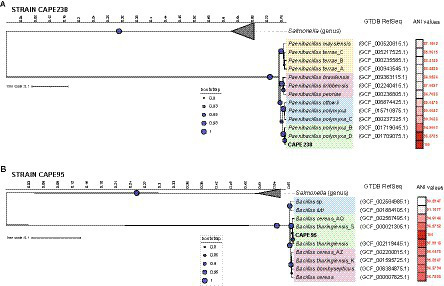
Phylogeny of the endophytes *Paenibacillus* sp. CAPE238 **(A)** and *Bacillus* sp. CAPE95 **(B)** isolated from *Tropaeolum majus* roots. The concatenated tree of 120 marker genes was constructed with the software GTDB-Tk, and metadata (GTDB RefSeq and ANI values) were added with the software iTOL v6.

The whole genomes of both strains were used to infer a more definitive taxonomic classification at the species level (Figure 1). Taxonomic assignment was initially performed using the software GTDB-Tk, which uses the Genome Taxonomy Database (GTDB) to identify 120 marker genes in the genome, infer and align the translated proteins in the database and construct the phylogenetic analysis. The results showed that the CAPE238 strain is closely related to *Paenibacillus polymyxa*, with an ANI value of 98.8% (above the 95% ANI threshold for the same species assumption; Figure 1A).

The CAPE95 strain was closely related to the *Bacillus cereus* group; however, the ANI values were not sufficient for classification at the species level using this method (Figure 1B). The CAPE95 strain was closely related to *Bacillus thuringiensis*, *B. cereus* and *B. bombysepticus* and exhibited an ANI > 95% (Figure 1B). To confirm the strain’s identity, both the cgMLST and rMLST tools were used, and showed that CAPE95 shares 100% similarity with *Bacillus thuringiensis*. Therefore, the targeted strains were reliably identified taxonomically as *Bacillus thuringiensis* (CAPE95) and *Paenibacillus polymyxa* (CAPE238).

#### 3.1.2 Functional annotation of the genomes of the CAPE95 and CAPE238 strains

**Figure 2:**
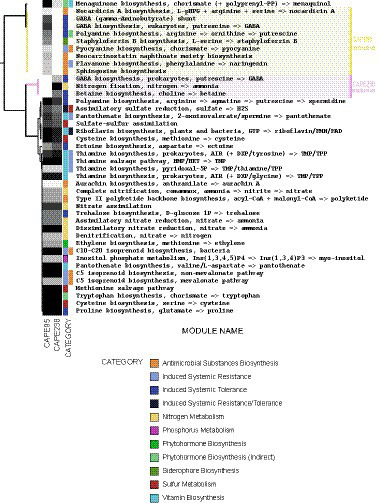
Functional prediction of the strains *Paenibacillus polymyxa* CAPE238 and *Bacillus thuringiensis* CAPE95. The metabolic pathways were annotated with KEGG software and plotted with Anvi’o. The heatmap shows the completeness of the pathways from 0% (white) to 100% (black).

To identify genes and pathways that could be related to the biofertilization mechanisms of the strains, metabolic predictions were performed. Figure 2 shows the metabolic pathways directly or indirectly associated with biofertilization found in both the CAPE238 and/or CAPE95 genomes through KEGG annotation. A total of 42 pathways were found and they were associated with at least one biofertilization trait. The following metabolic pathways were annotated in both strains’ genomes: biosynthesis of metabolites possibly related to induced systemic resistance (biotic stressor resistance) and/or induced systemic tolerance (abiotic stressor tolerance; 32.7%), biosynthesis of antimicrobial substances (19.2%), vitamin production (15.4%), nitrogen metabolism (11.5%), sulfur metabolism (9.6%), direct or indirect biosynthesis of phytohormones (7.7%), phosphorus metabolism (1.9%) and siderophore biosynthesis (1.9%) (Figure 2). All percentages correspond to the proportion of each metabolic pathway within the total number of pathways annotated.

Both strains showed pathways that could promote plant growth, such as the production of the vitamins pantothenate and thiamine, the biosynthesis of the phytohormone ethylene and the nutrient cycling pathways of inositol phosphate metabolism (related to phytate turnover), nitrification and denitrification (Figure 2). Indirectly, these strains could also promote growth by acting as biocontrol agents through the production of antimicrobial substances, such as polyketides (Figure 2). In contrast, the *P. polymyxa* CAPE238 strain showed the nitrogen fixation pathway, whereas *B. thuringiensis* CAPE95 had additional pathways related to antimicrobial biosynthesis, such as naringenin and nocardicin A (Figure 2).

Finally, Prokka annotation also provided additional information about genes present in both strains that could be related to salt stress tolerance (*deg*S and *deg*U), the production of siderophores (bacillibactin), surfactin (*srfA* and *srfD*) and other antimicrobial substances (AMS), such as bacilysin in CAPE95 and the lantibiotic paenibacillin in CAPE238. Therefore, we suggest that the CAPE95 and CAPE238 strains could be applied together as a biofertilizer since they harbor various shared and specific mechanisms for each strain that should work complementarily and promote plant growth.

### 3.2 *Tropaeolum majus* biofertilization with the consortium *Bacillus thuringiensis* CAPE95 and *Paenibacillus polymyxa* CAPE238

**Figure 3:**
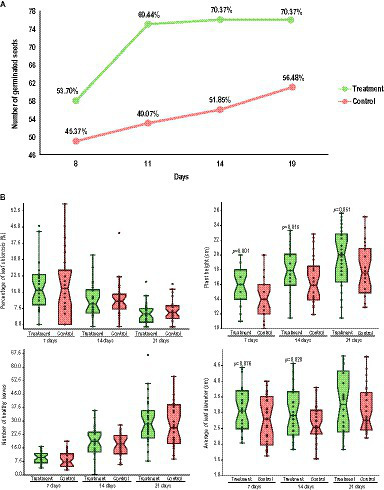
Biofertilization *in vivo* experiments in *Tropaeolum majus* with the co-inoculation of *B. thuringiensis* CAPE95 and *P. polymyxa* CAPE238. **(A)** Comparison of the germination index of treated (in green) and control (in red) seeds. **(B)** Comparison of growth parameters over time (7, 14, and 21 days) in seedlings in both conditions (treatment and control). The statistics were performed with a two-way ANOVA, considering *p* < 0.1, *p* < 0.05, and *p* < 0.01.

A 21-day experiment was performed to evaluate the capacity of the CAPE95 + CAPE238 consortium (treatment) to increase the germination rate of *T. majus* seeds. The results showed that while the treated seeds reached the peak germination rate in 11 days, the control seeds continued to germinate throughout the experiment (Figure 3A). By the 11th day, the germination rate of the treated seeds was 20.3% higher than that of the control, and by the 21st day, it was 13.9% higher (Figure 3A). The results of this test indicate that the treated seeds showed a higher germination rate and had a tendency to germinate faster when compared to the control.

Thirty days after the seed treatment, the grown seedlings were reinoculated with either the treatment (CAPE95 + CAPE238) or the control (sterile 1X PBS), and another 21-day experiment was performed. The average leaf diameter was significantly higher in the treated plants after 7 (*p* < 0.1) and 14 days (*p* < 0.05) and maintained the same tendency up to 21 days (Figure 3B). Moreover, the plant height was significantly higher in the treated plants throughout the whole experiment (7 days *p* < 0.01; 14 days *p* < 0.05 and 21 days *p* < 0.1; Figure 3B). The number of healthy leaves was slightly higher and the percentage of chlorosis was slightly lower in the treated plants throughout the experiment, even though these results were not statistically significant (Figure 3B). Therefore, it is possible to infer that the treatment is able to promote *T. majus* growth, especially by increasing plant height and leaf diameter.

### 3.3 Impact of the consortium *Bacillus thuringiensis* CAPE95 and *Paenibacillus polymyxa* CAPE238 treatment on the root bacteriome of *Tropaeolum majus* plants

At the end of the *in vivo* experiments in the greenhouse, the total DNA was isolated from the roots of 18 *T. majus* plants (nine for the control and nine for the treatment) and sequenced via 16S rRNA gene metabarcoding. A total of 1,165,555 sequences were obtained, and a positive control showed that the sequencing had an overall error rate of 0.0114. Moreover, a negative control was used to remove possible contaminants from the sequences, which were normalized to 27,365 per sample and resulted in a total of 722,701 sequences and 19,192 OTUs. The rarefaction curves indicate that the number of samples was enough to cover most of the local bacterial diversity, whereas the bacteriome of the plants treated with the consortium showed a higher diversity (OTU richness) when compared to the control (Supplementary Figure S1).

#### 3.3.1 Bacterial community diversity analyses

**Figure 4:**
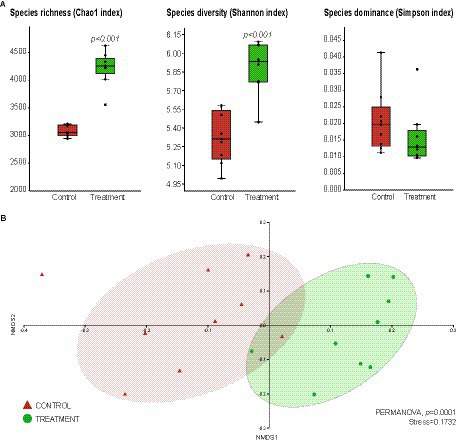
Impact of *Bacillus thuringiensis* CAPE95 and *P. polymyxa* CAPE238 co-inoculation on the *Tropaeolum majus* root bacterial community. The assessment of alpha-diversity **(A)** and beta-diversity **(B)** was conducted through a comparative analysis of Operational Taxonomic Units (OTUs) between the treatment (nine replicates, represented in green) and the control (nine replicates, represented in red). Sequences obtained from 16S rRNA gene metabarcoding were analyzed using Mothur to generate the OTU matrix. The graphs were plotted using PAST 4.02 software.

Alpha and beta-diversity analyses were performed to better understand the impact of the consortium inoculation on the root bacteriome of *T. majus* plants (Figure 4). The alpha-diversity analysis showed that the bacteriome of the treated plants had significantly higher species richness (Chao1 index, *p* < 0.001) and diversity (Shannon index, *p* < 0.001) when compared to the bacteriome of the control (Figure 4A). Moreover, the bacterial community of the control plants showed a higher dominance of species (Simpson index) when compared to the treatment, even though no significant differences were found (Figure 4A).

The beta-diversity was represented by NMDS, which revealed that the bacterial community from the treatment group differed significantly (PERMANOVA, *p* < 0.001) from that of the control (Figure 4B). These results indicate that the bacteriome of the treated plants was significantly different from that of the control and showed a higher diversity and richness of bacterial species.

#### 3.3.2 Bacterial community taxonomic structure

The bacteriome composition was accessed by taxonomically classifying the OTUs obtained from the sequences and calculating their relative abundance. The core bacteriomes of both the treated and control *T. majus* plants were dominated by the phyla Proteobacteria (control – 55% and treatment – 57%), Actinobacteria (control – 25% and treatment – 27%) and Firmicutes (control – 3.6% and treatment – 3.5%), with no significant differences in relative abundance observed between the treatment and control groups (Supplementary Figure S2). Moreover, the most abundant OTUs from the core bacteriome were associated with the genera *Streptomyces*, *Rhizomicrobium*, *Devosia*, and *Actinoallomurus* and the Xanthomonadaceae family (Supplementary Figures S3, S4).

**Figure 5:**
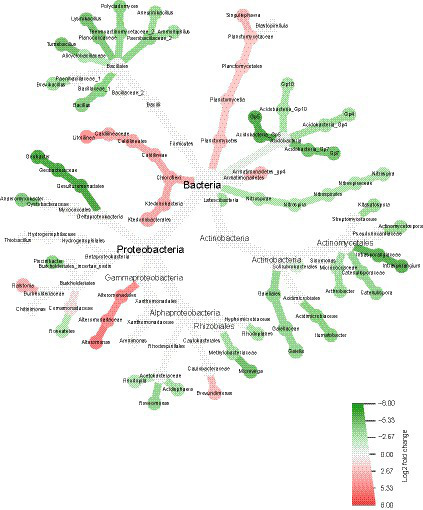
Relative abundance of taxa in the bacterial community of the treated plants (green) and the control plants (red). The heat tree generated with the Metacoder package in RStudio was filtered to show only significant enrichment differences (Wilcoxon test, *p* < 0.05).

To understand how the treatment impacted the *T. majus* plant bacteriome, a heat tree comparing the taxonomic abundance of taxa from each of the conditions (treatment and control) is shown in Figure 5. The heat tree was filtered to only show results with significant differences (Wilcoxon test, *p* < 0.05).

The bacterial community of the treated plants had several taxa enriched in the phyla Firmicutes, Acidobacteria, Actinobacteria and Nitrospirae compared to those in the control. In the phylum Firmicutes and Bacillales order, the genera *Ammoniphilus*, *Aneurinibacillus*, *Polycladomyces*, *Lysinibacillus*, *Tumebacillus*, *Brevibacillus*, and *Bacillus* were significantly enriched. Moreover, in the phylum Acidobacteria, the subgroups Gp5, Gp10, Gp4, and Gp7 were also enriched. In the phylum Actinobacteria, diverse taxa were enriched, such as the genera *Arthrobacter*, *Actinomycetospora*, and *Instrasporangium*. Finally, under the phylum Proteobacteria, several genera, such as *Anaeromyxobacter*, *Microvirga*, and *Geobacter*, were also enriched.

Comparatively, the bacteriome of the control plants showed enrichment of the phyla Chloroflexi and Planctomycetes, as well as the genera *Litorilinea* and *Singulisphaera*. In the Proteobacteria phylum, the genera *Ralstonia*, *Alteromonas*, and *Brevundimonas* were also enriched when compared to those in the treatment bacteriome. These results suggest that the bacterial community of the treated plants was enriched in taxa commonly associated with PGP when compared to the control, which could be indirectly helping to improve the plant growth observed in the previous experiments.

## 4 Discussion

The search for biofertilizers as a way to contain the use of chemical fertilizers that cause great negative impacts on the health of people, animals and the environment is growing worldwide. Chemical fertilizers can reduce soil fertility, alter the diversity of soil microorganisms and cause groundwater pollution. In this research, we evaluated the potential of two well-known plant growth-promoting bacteria (PGPB) to promote the growth of an underexplored medicinal and edible plant. To our knowledge, this is the first study testing the combination of *Bacillus thuringiensis* and *Paenibacillus polymyxa* as a biofertilizer and the first plant growth-promoting study of *Tropaeolum majus* plants.

By analyzing the genomes of the CAPE95 and CAPE238 strains, which are endophytes previously isolated from *T. majus* roots (Dal’Rio et al., 2022), we determined that they belonged to *B. thuringiensis* and *P. polymyxa*, respectively. Both species have been previously described as PGPB since they can colonize the soil-root interface and the endosphere of different plant species, produce metabolites that enhance plant fitness and elicit the plant innate immune response (Langendries and Goormachtig, 2021; Gomis-Cebolla and Berry, 2023). Endophytic colonization by these bacteria may occur by vertical or horizontal transmission, i.e., via the seed core microbiome throughout different generations or via recruitment from the external environment to the seed or plant, respectively (Langendries and Goormachtig, 2021; Gomis-Cebolla and Berry, 2023). Thus, *T. majus* plants may be colonized by the CAPE95 and CAPE238 strains in their endophytic microhabitats, which allows these PGPB to be isolated from external environmental stressors, such as UV light, which could contribute to plant growth promotion. Additionally, the ability of both species to colonize and promote the growth of different plant species at various developmental stages suggests that the application of *B. thuringiensis* CAPE95 and *P. polymyxa* CAPE238 as biofertilizers could benefit plants other than *T. majus*.

Both *B. thuringiensis* and *P. polymyxa* are widely explored because of their biotechnological potential. Strains belonging to *B. thuringiensis* are important agents for pest and phytopathogen control, especially because of the production of insecticidal and antimicrobial substances (Gomis-Cebolla and Berry, 2023). In addition to their relevance for agricultural applications, *B. thuringiensis* strains have also been explored due to the production of biomolecules with bioremediation potential, proteins with anticancer properties and clinically relevant antimicrobial peptides (Oliveira-Santos et al., 2023). Comparatively, *P. polymyxa* strains are distinguished biofertilizers that often show a variety of plant growth-promoting (PGP) traits, such as enhanced nutrient uptake by plants and phytohormone production, and may even act as biocontrol agents against phytopathogens (Langendries and Goormachtig, 2021). These strains may also produce secondary metabolites relevant for various industries, such as 2,3-butanediol and exopolysaccharides, which can be applied in waste management and agricultural, food and cosmetic industries (Langendries and Goormachtig, 2021). Considering the versatility of *B. thuringiensis* and *P. polymyxa*, genomic analysis of these strains can contribute to an enhanced awareness of their biotechnologically relevant molecules and possible applications.

After performing a genome function annotation, numerous genes and metabolic pathways associated with PGP were found. The biofertilization capacity of CAPE95 and CAPE238 relies on the production of metabolites that act as signaling molecules and can induce systemic resistance and/or tolerance in plants (Figure 2). The production of spermidine has been previously related to eliciting salt tolerance in *Arabidopsis thaliana* and *Zea mays* and can also promote plant growth by regulating ethylene levels in the plants (Xie et al., 2014; Chen et al., 2017). Furthermore, gamma-aminobutyric acid (GABA) biosynthesis is also known to upregulate the synthesis of pathogenesis-related (PR) genes in plants related to systemic acquired resistance, which protects the plant against phytopathogens and nematodes (Kim et al., 2019).

With respect to plant hormone production, tryptophan is a main precursor for the production of auxins through tryptophan-dependent pathways, such as indole-3-acetic acid, which promote root elongation, ramification and may improve seed germination (Batista et al., 2021). Vitamin production is an underexplored trait that also has a role in plant development, and we detected pathways related to the biosynthesis of pantothenate, thiamine, riboflavin and others. These vitamins can stimulate plant growth through different mechanisms, such as promoting shoot and root growth, favoring nitrogen fixation and increasing carbon assimilation (Palacios et al., 2014).

PGPB are also known to improve nutrient uptake by plants. In a previous study (Dal’Rio et al., 2022), *in vitro* PGPB screening tests revealed that the strains *B. thuringiensis* CAPE95 and *P. polymyxa* CAPE238 were able to produce siderophores and antimicrobial substances, solubilize and mineralize phosphate, while the *nifH* gene—related to nitrogen fixation capability—was detected only in the CAPE238 strain. Moreover, the production of indole-related compounds was observed only in the CAPE95 strain. We hereby confirm that metabolic pathways possibly related to these traits were also found in the genome.

To investigate the ability of the strains to promote growth *in vivo*, a consortium of the endophytes *B. thuringiensis* CAPE95 and *P. polymyxa* CAPE238 was inoculated into the seeds and seedlings of *Tropaeolum majus* plants. The greenhouse experiments showed that the consortium CAPE95 + CAPE238 was able to accelerate seed germination, thus guaranteeing a higher number of germinated seeds when compared to the control (Figure 3). Moreover, seedlings treated with the consortium also showed higher plant height and leaf diameter, thus demonstrating that the treatment promoted plant growth (Figure 3). Considering that the leaves of the plant can be commercialized, this is a promising result. Since *T. majus* is a perennial plant and the edible flowers are also commercialized, the next steps should include the analysis of the impact of the consortium on the number of flowers on older plants.

The alpha and beta-diversity analyses of *T. majus* plants showed that the bacterial community of the treated plants was significantly different from the bacterial community of the control, whereas the bacterial community of the treated plants showed a higher diversity and richness (Figure 4). These results indicate that *B. thuringiensis* CAPE95 and *P. polymyxa* CAPE238 co-inoculation was able to enhance the functional diversity in the environment, thus impacting plant growth. The synergistic effects of different bacterial taxa could help improve nutrient availability in plants and outcompete potential phytopathogenic microorganisms (Wang et al., 2021).

A variety of taxa were significantly enriched in the bacteriome of the treated plants. Within the Proteobacteria phylum, the genera *Microvirga*, *Anaeromyxobacter*, and *Geobacter* have been previously described as diazotrophic bacteria (Jiménez-Gómez et al., 2019; Masuda et al., 2020). The genus *Nitrospira*, which is in the phylum Nitrospirae, is related to an increase in nitrogen uptake by plants (Vijayan et al., 2021). Although the relationships of Acidobacteria subgroups (Gp4, Gp5, Gp7, Gp10) with plants are not well understood due to the fastidious nature of these bacteria, Kalam et al. (2017) reported that tomato plants treated with PGPB enhanced the Acidobacteria population over time and suggested that they could also have a role in plant growth promotion.

Within the phylum Actinobacteria, *Arthrobacter* is a versatile genus that is associated with the capacity to degrade pesticides in soil and promote plant growth under salt stress (Safdarian et al., 2019; Wang et al., 2023). Moreover, the genera *Intrasporangium* and *Actinomycetospora* have both been previously described as plant endophytes, but their potential plant growth traits remain unexplored (El-Shatoury et al., 2013; Kaewkla and Franco, 2019).

Interestingly, a higher abundance of various taxa within the order Bacillales was observed in the bacterial community of the treated plants. Among the taxa enriched, the genus *Lysinibacillus* possesses insecticidal properties and can produce phytohormones (Pantoja-Guerra et al., 2023). Furthermore, *Tumebacillus* sp. could enhance phosphorus uptake by plants, and *Brevibacillus* sp. can promote plant growth under abiotic stresses, such as in the presence of heavy metals (Vasconcellos et al., 2021; Wani et al., 2023).

In contrast, the bacteriome of the control plants was enriched with the genera *Litorilinea* and *Singulisphaera*, which were described to be present in the rhizosphere of halophytes from highly saline soils and linked to nutrient cycling (Mukhtar et al., 2018). Moreover, the genus *Ralstonia* includes economically relevant phytopathogenic species, such as *Ralstonia solanacearum*, and its presence in the *Tropaeolum majus* root bacterial community has already been described previously (Dal’Rio et al., 2022). Since the plants in this study did not show symptoms of the disease caused by phytopathogenic *Ralstonia* –, i.e., yellowing and wilting of the leaves—the relationship between the *Ralstonia* genus and *Tropaeolum majus* plants remains unclear. To our knowledge, no commensal relationship between this genus and plants has been described before.

Finally, the results suggest that the CAPE95 + CAPE238 consortium positively impacts the *Tropaeolum majus* bacterial community by enhancing the richness and diversity of potential synergistic PGPB. Since the genomes of the strains harbor a variety of PGP traits, further studies will elucidate which metabolic pathways are enriched after plant treatment. Furthermore, we hope to evaluate their growth promoting ability in different economically relevant plant species, in the hopes of developing bioproducts for biofertilization in the future.

## 5 Conclusion

Two endophytes previously isolated from *Tropaeolum majus* roots were taxonomically assigned as *Bacillus thuringiensis* CAPE95 and *Paenibacillus polymyxa* CAPE238. The two strains were co-inoculated for the first time in *T. majus*, and they were able to accelerate and increase seed germination and promote *T. majus* seedling growth. Genome mining of these strains revealed that they showed different plant growth-related genes and metabolic pathways, such as those involved in the biosynthesis of metabolites that elicit plant immunity against biotic and abiotic stresses. The bacterial community of plants treated with the consortium was significantly different from the bacteriome of the control and showed a higher diversity and richness of bacterial species. Moreover, consortium inoculation increased the diversity of other potential PGPB in the *Tropaeolum majus* bacteriome. The data presented here demonstrate that the bacterial consortium has great potential for application as a biofertilizer in *T. majus* and that its use could be further extended to other plant species.

## Data availability statement

The datasets presented in this study can be found in online repositories. The names of the repository/repositories and accession number(s) can be found here: https://www.ncbi.nlm.nih.gov/genbank/, JAWPHG000000000 (CAPE95) and JAWPHF000000000 (CAPE238), https://www.ncbi.nlm.nih.gov/genbank/, BioProject PRJNA1033832.

## Author contributions

ID’R: Conceptualization, Data curation, Formal analysis, Investigation, Methodology, Writing – original draft, Writing – review & editing. EL: Formal analysis, Investigation, Methodology, Writing – review & editing. KS: Formal analysis, Investigation, Methodology, Writing – review & editing. AR: Conceptualization, Funding acquisition, Resources, Supervision, Writing – review & editing. LS: Conceptualization, Funding acquisition, Project administration, Supervision, Writing – original draft, Writing – review & editing.

## Footnotes

^1^https://itol.embl.de

^2^https://pubmlst.org/

^3^https://mothur.org/wiki/miseq_sop/
