# Supplementary material for: Co-inoculation of the endophytes Bacillus thuringiensis CAPE95 and Paenibacillus polymyxa CAPE238 promotes Tropaeolum majus L. growth and enhances its root bacterial diversity
Source: Front Microbiol. 2024 Feb 23;15:1356891. doi: 10.3389/fmicb.2024.1356891 (PMC10996857; doi:10.3389/fmicb.2024.1356891)
Supplement: Supplementary file 1 [file Data_Sheet_1.docx]

**Co-inoculation of the endophytes *Bacillus thuringiensis* CAPE95 and *Paenibacillus polymyxa* CAPE238 promote *Tropaeolum majus* L. growth and enhance its root bacterial diversity**

Isabella Dal’Rio^1,2^, Eliene dos Santos Lopes^1^, Karen Caroline Ferreira Santaren^1^, Alexandre Soares Rosado^1,2^ and Lucy Seldin^1^*.

**Supplementary Material**

**Table S1** – QUAST assembly report of the genome of the strains *Paenibacillus polymyxa* CAPE238 and *Bacillus thuringiensis* CAPE95. The assembly was performed using the software Unicycler.

| **Assembly** | **CAPE238** | **CAPE95** |
| --- | --- | --- |
| # contigs (>= 0 bp) | 118 | 71 |
| # contigs (>= 1000 bp) | 46 | 40 |
| # contigs (>= 5000 bp) | 29 | 30 |
| # contigs (>= 10000 bp) | 27 | 28 |
| # contigs (>= 25000 bp) | 23 | 26 |
| # contigs (>= 50000 bp) | 17 | 22 |
| Total length (>= 0 bp) | 5,767,258 | 5,655,448 |
| Total length (>= 1000 bp) | 5,740,151 | 5,644,286 |
| Total length (>= 5000 bp) | 5,705,791 | 5,617,995 |
| Total length (>= 10000 bp) | 5,690,648 | 5,604,286 |
| Total length (>= 25000 bp) | 5,626,781 | 5,574,099 |
| Total length (>= 50000 bp) | 5,429,246 | 5,407,998 |
| # contigs | 68 | 49 |
| Largest contig | 1,313,384 | 624,373 |
| Total length | 5,755,814 | 5,650,043 |
| GC (%) | 45.53 | 34.85 |
| N50 | 458,543 | 378,267 |
| N75 | 228,049 | 175,159 |
| L50 | 4 | 6 |
| L75 | 9 | 11 |
| # N's per 100 kbp | 0.00 | 0.00 |
| Complete BUSCO (%) | 99.32 | 99.32 |
| Partial BUSCO (%) | 0.68 | 0.68 |

**Figure S1**. Rarefaction curves of each replicate from the 16S rRNA gene metabarcoding analysis of *T. majus* roots. Replicates CB3, CB8, CB9, CC2, CC7, CC9, CF2, CF6 and CF7 represent the control plants and TA10, TA3, TA8, TD1, TD5, TD6, TE4, TE6 and TE7 represent the treated plants.

**
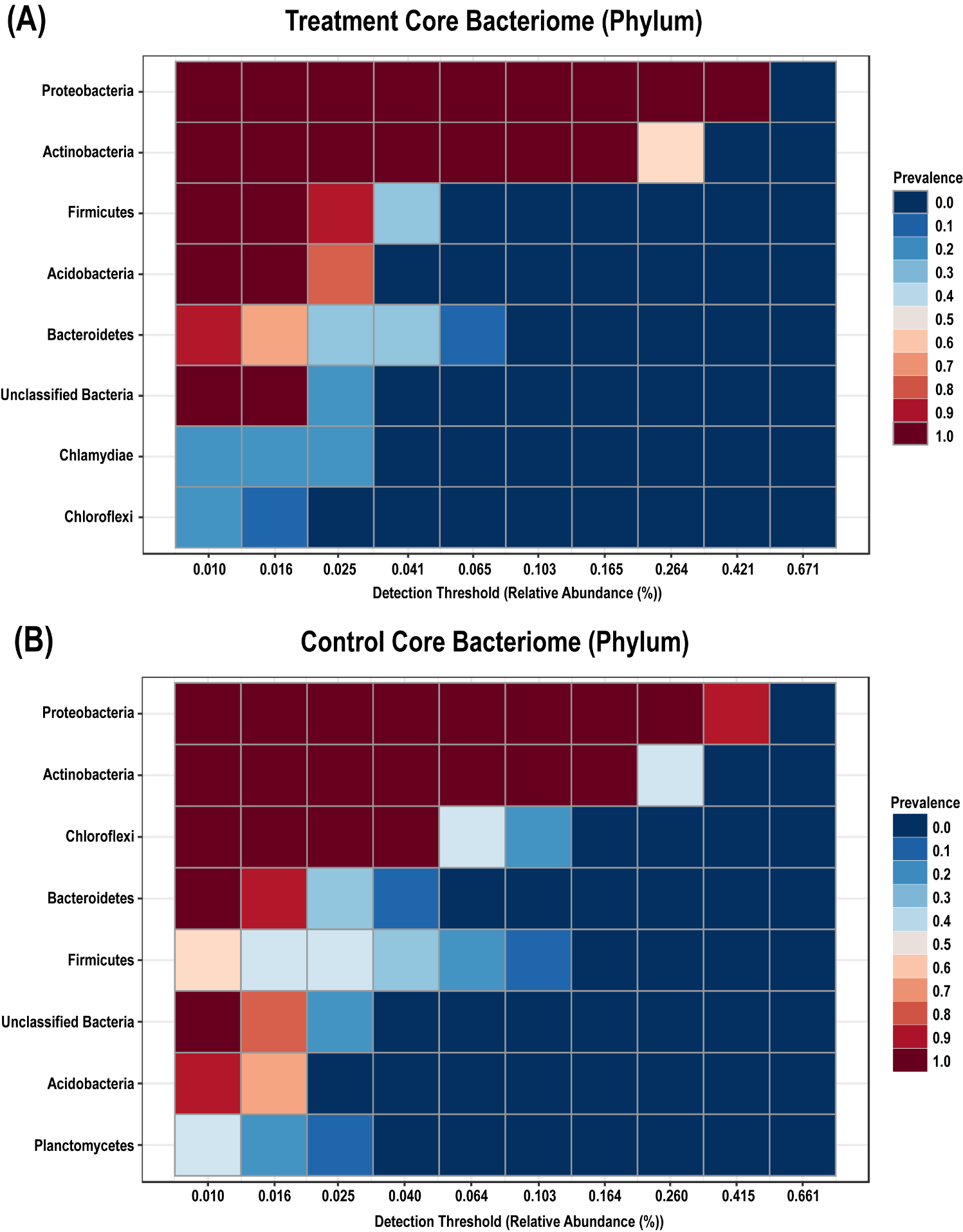
**

**Figure S2.** Core bacteriome (phylum level) of the nine replicates of *T. majus* plants treated with the consortium (A) and the control (B). The 16S rRNA gene metabarcoding sequences were used to plot the heatmaps using the tool MicobiomeAnalyst 2.0.

**
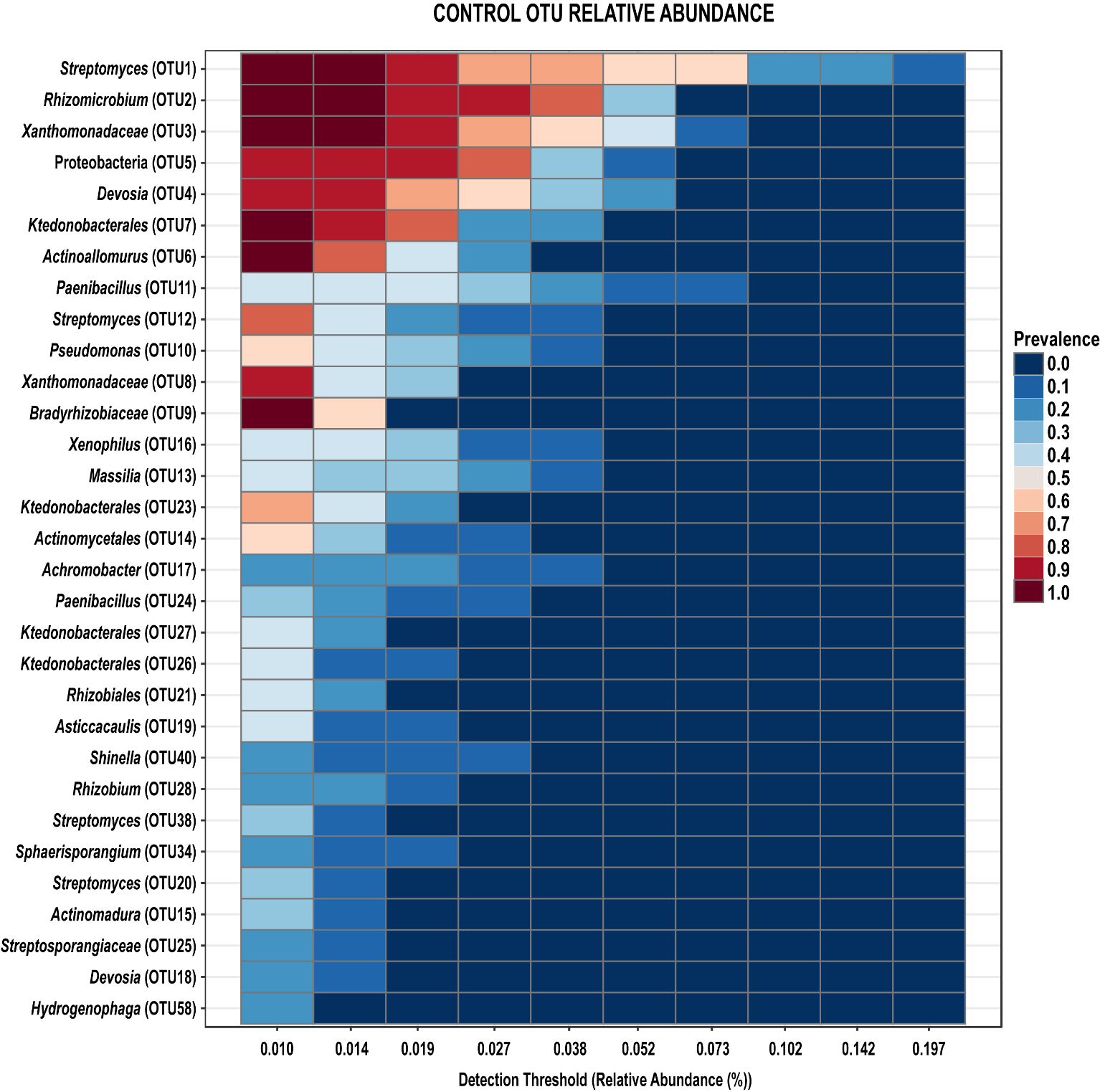
**

**Figure S3.** Core bacteriome (OTU) of the nine replicates of the control *T. majus* plants. The 16S rRNA gene metabarcoding sequences were used to plot the heatmaps using the tool MicobiomeAnalyst 2.0.

**
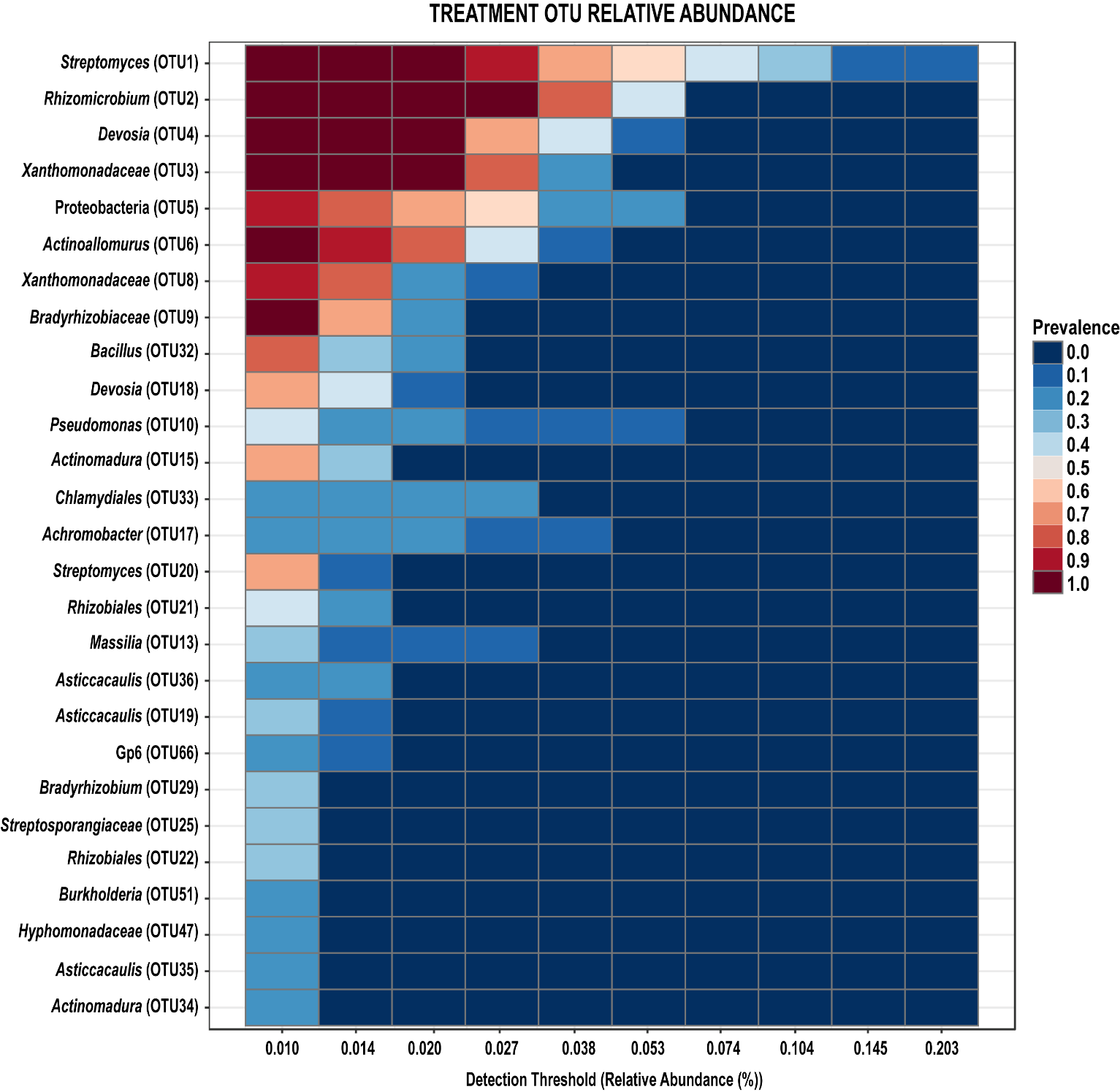
**

**Figure S4.** Core bacteriome (OTU) of the nine replicates of the *T. majus* plants treated with the consortium. The 16S rRNA gene metabarcoding sequences were used to plot the heatmaps using the tool MicobiomeAnalyst 2.0.
